# An Intensified Esterification Process of Palm Oil Fatty Acid Distillate Catalyzed by Delipidated Rice Bran Lipase

**DOI:** 10.1100/tsw.2006.213

**Published:** 2006-09-07

**Authors:** Fui Chin Chong, Beng Ti Tey, Zanariah Mohd. Dom, Nordin Ibrahim, Russly Abd. Rahman, Tau Chuan Ling

**Affiliations:** ^2^ Department of Process and Food Engineering, Faculty of Engineering University Putra Malaysia, 43400 Serdang, Selangor, Malaysia; ^1^ Department of Chemical and Environmental Engineering, Faculty of Engineering University Putra Malaysia, 43400 Serdang, Selangor, Malaysia

**Keywords:** esterification, palm oil fatty acid distillate, acylglycerols, packed bed reactor, rice bran lipase

## Abstract

An intensified esterification process was operated by circulating 10 l of reaction mixtures, consisting of palm oil fatty acid distillate (PFAD) and glycerol in hexane, through a packed-bed reactor (PBR) filled with 10 kg of delipidated rice bran lipase (RBL). The influence of the process parameters, such as reaction temperature and type of water-removal agent, on the performance of this intensified esterification process were investigated. The highest degree of esterification (61%) was achieved at a reaction temperature of 65°C, using silica gels as the water-removal agent. Thin-layer chromatography (TLC) analysis showed that the major composition of the esterified product was diacylglycerol.

## INTRODUCTION

Medium-chain acylglycerols, a mixture of mono-, di-, and triacylglycerols, are important ingredients for the food, pharmaceutical, and cosmetic industries. In addition, diacylglycerol is widely used in the formulation of traditional foods and in obesity-management diets[1] Medium-chain acylglycerols can be produced from the esterification of free fatty acid and glycerol. This esterification process is usually catalyzed by microbial lipase. The application of plant-sourced lipase for this purpose is limited[2]. Plant-sourced lipase is usually left unutilized as waste in the food-processing industry[3]. For instance, rice bran, a by-product from the rice-processing industry, is usually utilized as animal feed. The stability of native rice bran lipases (RBL) in organic solvent and high temperature is low. However, after a delipidation process, its stability was substantially improved[4]. This delipidation process has permitted the use of RBL as a biocatalyst in the esterification of palm oil fatty acid distillate (PFAD) and glycerol to produce medium-chain acylglycerols[4]. The PFAD is a by-product from the local palm oil refineries, and it has been previously used as a source of free fatty acids for the production of acylglycerol by other researchers[4,5].

The rates of enzymatic reactions usually increase with the reaction temperature and can be described by Arrhenius equation[6,7]. On the other hand, the stability of the enzymes is affected by the surrounding temperature. Extreme temperature will cause enzyme denaturation and, thus, loss of catalytic activity. The optimal temperature for most immobilized lipases is in the range of 45–65ºC[5,8]. PFAD is in crystal form at temperatures lower than 45ºC[5]; hence, the lowest reaction temperature for the esterification process involving PFAD used was 45ºC. As a result, the lipases used for this purpose have to be stable at temperatures higher than 45ºC.

Water generated during the esterification reaction has to be removed by the addition of water-removal agents[9,10] or by lowering the pressure of the reaction vessel[11]. In this study, the water produced by the esterification reaction was removed by circulating the reaction mixture through a water-removal column, which was filled with a water-removal agent external to the packed-bed reaction column. Typical water-removal agents frequently used include molecular sieves[9] and silica gels[12]. Maintaining a low water content in the reaction mixture ensures that undesirable hydrolysis reactions are suppressed, thereby permitting high esterification yields[13]. Packed-bed reactors (PBRs) are commonly used in the large-scale intensified process due to the nature of the low shear stress they exhibit. Higher shear stress induced by the agitation in a stirred tank reactor would cause the breakage of immobilized enzyme particles[10]. Another advantage of PBRs includes the increased densities of immobilized RBL that can be loaded into the reactor, thereby permitting process intensification.

In this study, we developed an intensified esterification process for the production of medium-chain acylglycerols, especially diacylglycerol. A PBR complete with a heating system to control the reaction temperature and a water-removal system was used for the esterification process of PFAD and glycerol. The PBR was packed with delipidated RBL, which acts as a biocatalyst for the esterification process. The process parameters that can influence the performance of PBR, temperature and the type of water-removal agent, were investigated. Both PFAD and RBL used in the present study are sourced from local food-processing industries. The utilization of these food-processing industry by-products for the production of medium-chain glycerides has an important impact on the local environment and economy.

## MATERIALS AND METHODS

### Materials

Materials including glycerol, molecular sieves, silica gel, and iodine were obtained from Sigma-Aldrich Inc. (U.S.). Technical grade n-hexane was obtained from Kofa Chemical Co. (Malaysia). The PFAD was supplied by Golden Jomalina Food Industries Sdn Bhd (Malaysia). Rice bran was first delipidated by dispersing in n-hexane for 1 h to remove the rice bran oil. The oil and n-hexane was filtered out and the depidated rice bran was dried for 6 h. The dried delipidated rice bran was then immersed in buffer A (0.05 *M* phosphate, pH 8) for 1 h at room temperature (25ºC). The delipidated RBL was then filtrated from the buffer solution and oven dried (40ºC for 5 days). The delipidated RBL was stored at –20°C for subsequent use.

### Intensified Esterification Process

The esterification of PFAD and glycerol catalyzed by immobilized RBL was performed in a PBR designed and fabricated in house (Figs. 1 and 2). The temperature of the PBR was controlled and maintained by the heating system and water jacket installed. In addition, a water-removal column filled with water-removal agent was used to remove the reaction water. The reactor was designed for down-flow in semi-continuous mode of operation. The reaction mixture (10 l), consisting of 2.0 kg of PFAD, 0.335 kg of glycerol, and 4.0 kg of hexane, was first loaded into the feeding tank of the PBR. The mixture was preheated to reach the reaction temperature (45, 55, and 65ºC). It was then pumped at a flow rate of 20 l/min to the PBR and

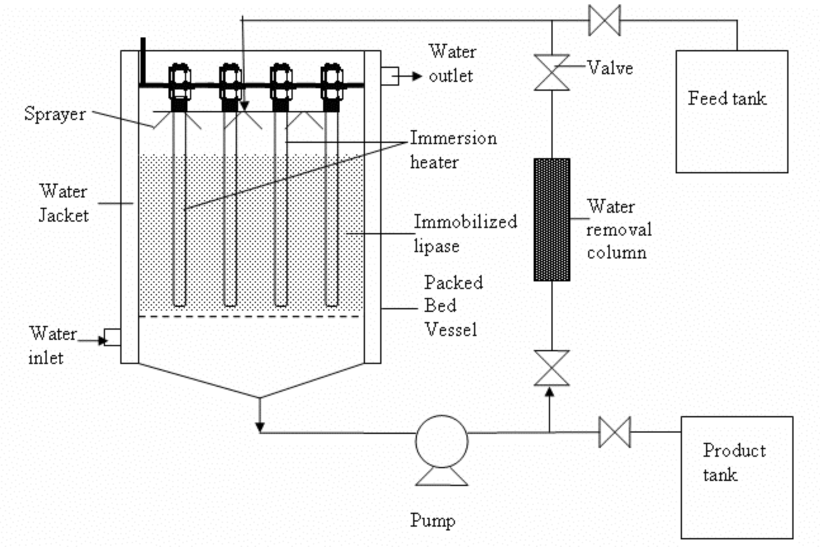

**FIGURE 1.** Schematic diagram of the setup of PBR use for the esterification process.

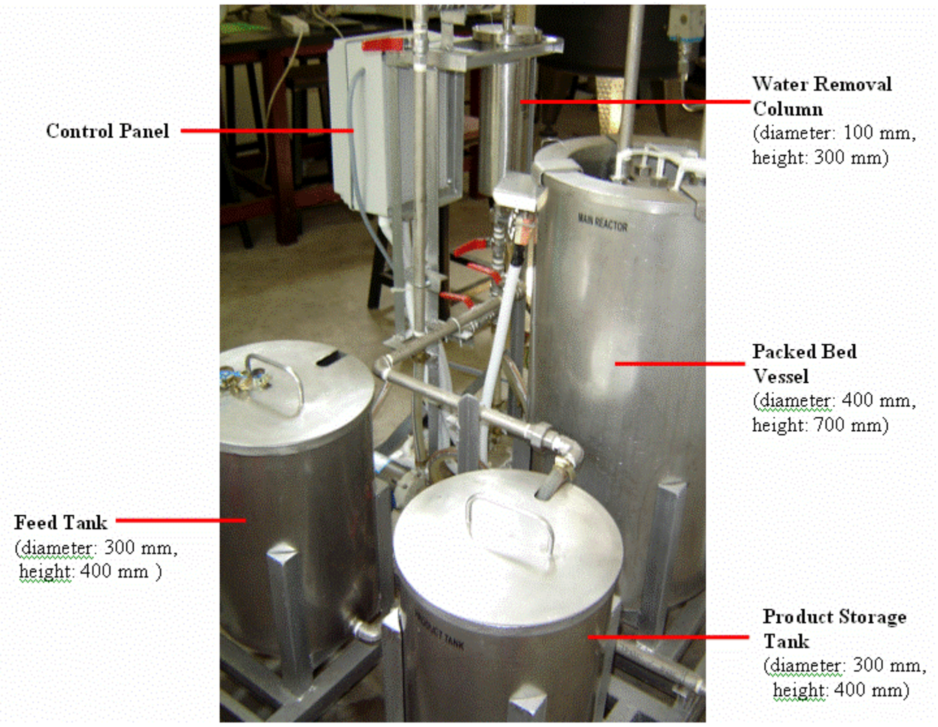

**FIGURE 2.** A photograph of the in-house-designed PBR used in this study. The vessels are built from stainless steel (SS 304).

sprayed over the surface of the delipidated RBL bed. The estimated residence time for the reactant in the PBR was about 1 min. The reaction mixture collected at the bottom of the vessel and was then recirculated back to the top of the PBR via the water-removal column at the same flow rate. The estimated residence time of the partially esterified product in the water-removal column was about 0.1 min. The recirculation of the partially esterified mixture was continued until a satisfactory degree of esterification was achieved. The esterified product was then stored in the product tank (Fig. 2). Silica gels and molecular sieves were investigated as water-removal agents to prevent the reverse hydrolysis of esterified product caused by the reaction water.

## Sample Analysis

Sample of reaction product was first titrated with 0.5 *M* NaOH using phenolphthalein to determine an endpoint. The titration endpoint, which showed the amount of fatty acid reacted, was used to calculate the degree of esterification. All the sample analyses were performed in triplicate. The degree of esterification was calculated based on the following equations:

$$\text{Amount of esterified free fatty acid}, \mu\text{mol} = (V_c - V_s) M \times 1000 \quad (1)$$

$$\text{Degree of esterification (ED)}, \% = \frac{\text{Amount of esterified free fatty acid}, \mu\text{mol}}{\text{Amount of free fatty acid in control reaction}, \mu\text{mol}} \times 100\% \quad (2)$$

where $V_c$ = volume of NaOH used for unesterified reaction mixture, ml; $V_s$ = volume of NaOH used for the sample, ml; M = molarity of NaOH solution.

Reaction products (mono-, di-, and triacylglycerol) were identified by thin-layer chromatography (TLC) method as previously described by Sonwalkar et al.[12]. TLC plates (4 cm × 14 cm) were precoated with silica gel (Sigma-Aldrich Inc., U.S.) and activated by heating at 105°C for 30 min. The samples were then applied onto the bottom of the plate.

The plate was heated briefly (10–15 sec) on a hot plate at 90°C to evaporate the water. The samples were spotted directly on the plate with authentic standards of mono-, di-, and triolein (Nu-Check-Prep, U.S.). The spotted plate was then developed in a solvent mixture containing chloroform:acetone:methanol (90:8:2 v/v/v). Spots of each glyceride were visualized by staining with iodine vapor.

## RESULTS AND DISCUSSION

The esterification activity of immobilized RBL in the PBR increased as the reaction temperature increased from 45 to 65ºC (Fig. 3). Maximum esterification was achieved in temperature order of 65, 55, and 45˚C. The degree of esterification at each temperature was 61, 36, and 19%, respectively. The lowest temperature used in this study was 45ºC due to the fact that the source of free fatty acids used in this study, PFAD, crystallizes at temperatures below 45ºC[4].

As the temperature increases, the PFAD begins to liquidize and the viscosity of the reaction mixture decreases. Therefore, there is a greater degree of interaction between the substrates and enzyme molecule at higher temperature, which promotes the esterification reactions. Also, the rate of water evaporation is higher at higher temperature, which may help to reduce the reverse hydrolysis reactions of esterified products. In our previous study in shake flasks[4], we found that the rate of esterification was reduced as the temperature increased to 75ºC. Hence, 65˚C was the maximum reaction temperature investigated. The results of the present study agreed with those reported by Lo et al.[5] and Watanabe et al.[11]. The intensity of the chromatograms shown in Fig. 4 has further suggested that the degree of esterification was indeed affected by the reaction temperature. The TLC also showed that the major component of the esterified product was diacylglycerol.

The water content in the reaction mixture must be maintained at low levels in order to suppress the undesired hydrolysis of the reaction product[11]. One molecule of water is produced when one molecule of acid is esterified. To favor the esterification reaction, the water produced must be removed from the medium as fast as possible[5]. In this study, water generated during the reaction was removed by the water-removal agent contained within the water-removal column (Fig. 1). The performance of two types of water-removal

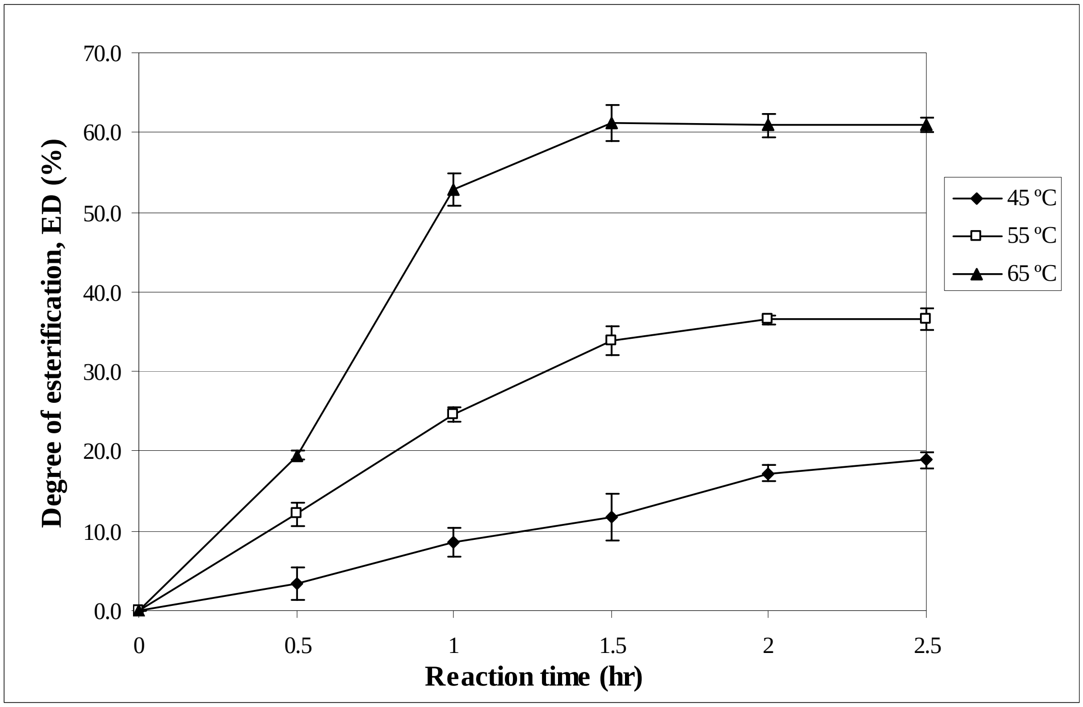

**FIGURE 3.** Effect of temperature on the performance of intensified esterification in a PBR. The reaction mixture consists of 2.0 kg PFAD, 0.336 kg glycerol (at a molar ratio of 2.0), and 4.0 kg hexane. The reaction water was removed by the silica gel (1 kg) filled in the water-removal vessel. The PBR was filled with 10 kg of immobilized RBL. Samples were tested in triplicate.

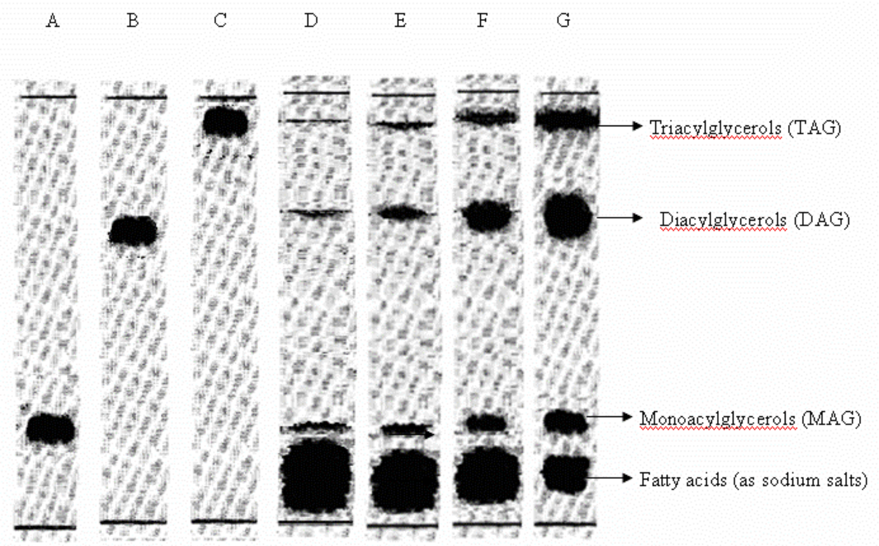

**FIGURE 4.** Chromatograms showing the fractionation of esterified product by TLC. Lane A, Lane B, Lane C are the standards of monoacylglycerol (MAG) (Rf = 0.26), diacylglycerol (DAG) (Rf = 0.72), and triacylglycerol (TAG) (Rf = 0.98), respectively. Lane D: the control reaction without the presence of lipase. Lane E, Lane F, and Lane G: the optimum esterification product fractions from reaction at 45, 55, and 65ºC, respectively [average Rf value: MAG (0.25), DAG (0.75), and TAG (0.96)].

agent, molecular sieves and silica gels, were investigated in this study (Fig. 5). The maximum degrees of esterification were 61, 58, and 8% for silica gel, molecular sieves, and control, respectively. The performance of both water-removal agents was identical during the initial stage. Minor differences were observed after 1 h of reaction. Similar observations were also obtained in the shake flask experiments[4].

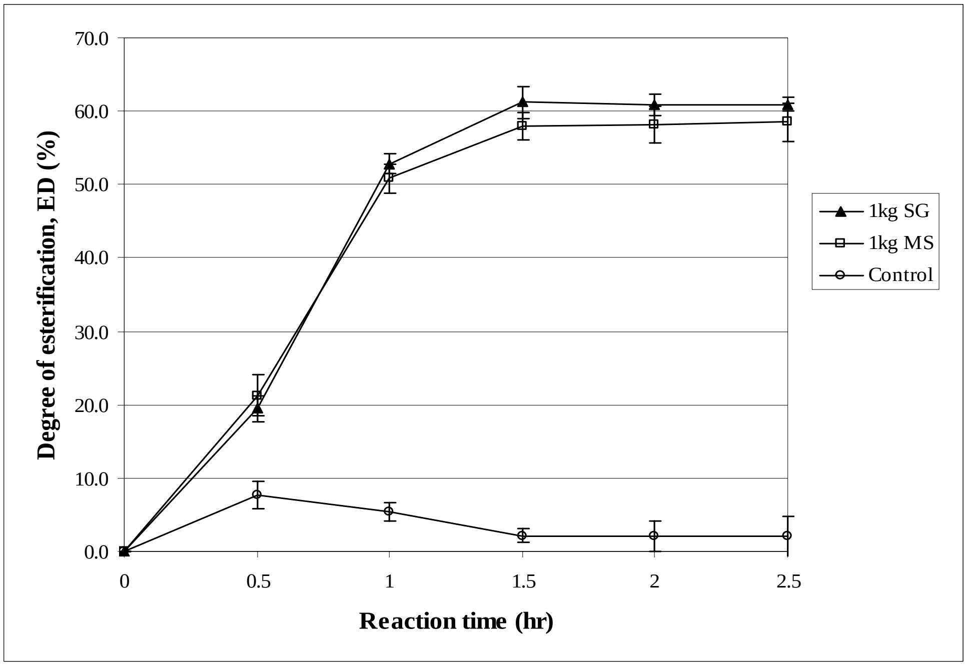

**FIGURE 5.** The effect of water-removal agents on the performance of intensified esterification in a PBR at 65ºC. The reaction mixture consisted of 2.0 kg PFAD, 0.336 kg glycerol (at molar ratio of 2.0), and 4.0 kg hexane. The water-removal column was filled with 1 kg silica gel (SG) or same amount of molecular sieve (MS). For the control experiment, the water-removal column was left empty. The PBR was filled with 10 kg of immobilized RBL. Samples were tested in triplicate.

Silica gel is commonly used as a water-removal agent in the esterification process[10,14]. It is a hard, translucent material made from amorphous silicate. The function of silica gel when adsorbing water is much like a tiny sponge with millions of microscopic pores. The water molecule is first adsorbed to the smallest pore, then followed by larger pore as the humidity increases. As a result, silica gel adsorbs more at higher humidity levels[15]. Beside, it has been suggested that silica gel has the ability to increase the liquid-liquid interface of the system (i.e., the interfacial surface area between hydrophilic glycerol and a hydrophobic organic solvent), allowing better adsorption of water molecules, thus improving the conversion of the fatty acid[14]. Kwon et al.[16] reported that a significantly enhanced fatty acid conversion is achieved by immobilizing glycerol onto silica gel in a lipase-catalyzed formation of diacylglycerol in n-hexane. The increased interfacial area between the hydrophilic glycerol and the hydrophobic organic solvent is suggested to be responsible for the higher esterification rate, in addition to its water-removal function[16]. A molecular sieve is a crystalline metal alumina silicate made from zeolite compound. It has a uniform pore structure, which allows it to separate molecules by size. Molecules are adsorbed to it based on polarity differences due to electrostatic attraction. Water molecules adsorb strongly to molecular sieves because of their higher degree of polarity and the right molecular size for entrance into the sieve's pores. Unlike silica gel, the water adsorption capacity of molecular sieves is lower at higher humidity levels[15]. This may explain the slightly difference in the degree of esterification at higher humidity conditions (after 1 h of reaction time) for both experiments using silica gel and molecular sieves as water-adsorption agents (Fig. 5).

The highest degree of esterification achieved in this study was only 61%; hence, there is still room to improve it. The ratios of RBL and water-removal agent to the reaction mixture used in this study have been previously optimized under shaked flask conditions[4]. Thus, further improvement may be achieved by the optimization of physical conditions, such as flow distribution in PBR and water-removal column. The effect of residence time of the reactants in PBR on the degree of esterification has been investigated by Watanabe et al.[10]. They found that the increase in residence time of the reactants in PBR has caused a decrease in the yield of diacylglycerol due to the undesired hydrolysis of the diacylglycerol as a result of insufficient water removal. However, it was not possible for us to investigate the effect of residence time of reactants in PBR and water-removal column in this study due to the system setup. The pumps used in the PBR system in this study are constant-flow-rate magnetic pumps. Hence, the flow rate of reactants of this system was not a controllable variable. Other factors affecting the performance and the scale up of this PBR system are the efficiency of mass and heat transfer in the PBR.

## CONCLUSION

We have successfully developed an intensified esterification process for the production of medium-chain acylglycerols, especially diacylglycerol, using by-product from local food-processing industries (PFAD and RBL). The highest degree of esterification (61%) was achieved at a reaction temperature of 65ºC, using silica gels as the water-removal agent. This value is comparable to that reported by other researchers. Wanatabe et al.[10] reported a value of 69% after a 2-h reaction in a PBR using reduced pressure to remove the reaction water. TLC analysis showed that the major composition of the esterified product was diacylglycerol.

## ACKNOWLEDGMENTS

This study was supported by IRPA project IRPA grant 09-02-04-0906-EA001 from the Ministry of Science, Technology and Innovation of Malaysia. We would like to thank Bernas Sri Tiram Jaya, Kuala Selangor, Malaysia for the fresh rice bran used in this study. The critical comments from the two reviewers were highly appreciated.
